# Dendritic cell analysis in primary immunodeficiency

**DOI:** 10.1097/ACI.0000000000000322

**Published:** 2016-10-17

**Authors:** Venetia Bigley, Dawn Barge, Matthew Collin

**Affiliations:** a Human Dendritic Cell Laboratory, Institute of Cellular Medicine, Newcastle University; b Blood Sciences Flow Cytometry Laboratory, Newcastle upon Tyne Hospitals NHS Foundation Trust, Newcastle upon Tyne, UK

**Keywords:** dendritic cell, GATA binding protein 2, immunodeficiency, interferon regulatory factor 8, monocyte

## Abstract

**Purpose of review:**

Dendritic cells are specialized antigen-presenting cells which link innate and adaptive immunity, through recognition and presentation of antigen to T cells. Although the importance of dendritic cells has been demonstrated in many animal models, their contribution to human immunity remains relatively unexplored *in vivo*.

Given their central role in infection, autoimmunity, and malignancy, dendritic cell deficiency or dysfunction would be expected to have clinical consequences.

**Recent findings:**

Human dendritic cell deficiency disorders, related to GATA binding protein 2 (GATA2) and interferon regulatory factor 8 (IRF8) mutations, have highlighted the importance of dendritic cells and monocytes in primary immunodeficiency diseases and begun to shed light on their nonredundant roles in host defense and immune regulation *in vivo*. The contribution of dendritic cell and monocyte dysfunction to the pathogenesis of primary immunodeficiency disease phenotypes is becoming increasingly apparent. However, dendritic cell analysis is not yet a routine part of primary immunodeficiency disease workup.

**Summary:**

Widespread uptake of dendritic cell/monocyte screening in clinical practice will facilitate the discovery of novel dendritic cell and monocyte disorders as well as advancing our understanding of human dendritic cell biology in health and disease.

## INTRODUCTION

Much of our knowledge of dendritic cell biology is inferred from mouse models and human in-vitro systems, which may not reflect the steady state and provide a limited understanding of host defense in the intact human. The analysis of dendritic cells in primary immunodeficiency disease (PID) has provided new diagnostic tools and revealed new clinical syndromes in addition to providing a unique opportunity to probe the function of human dendritic cells *in vivo*.

In this review, we explore what is known about dendritic cells and monocytes in PID, highlight the recently described dendritic cell deficiency syndromes related to GATA binding protein 2 (GATA2) and interferon regulatory factor 8 (IRF8) mutations, suggest a practical solution to dendritic cell analysis in clinical practice, and speculate how best to further our understanding of dendritic cells in PID and immunity in general. 

**Box 1:**
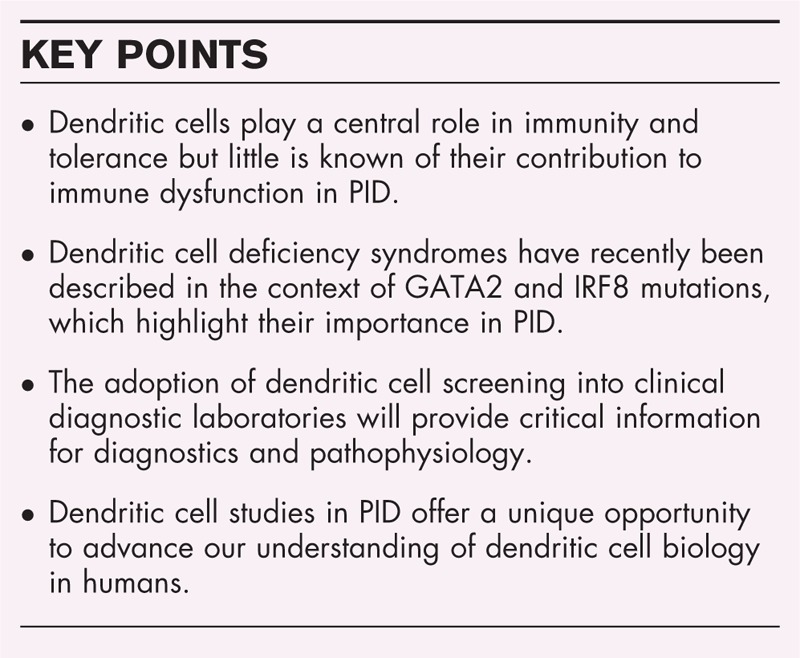
no caption available

## HUMAN DENDRITIC CELL BIOLOGY

Dendritic cells are bone marrow derived, specialized antigen-presenting cells (APCs), positioned within the immune system to bridge innate and adaptive immunity [1]. They are present in almost all tissues wherein they detect pathogens and process extracellular and intracellular antigen for presentation to T cells in the context of major histocompatibility complex (MHC) molecules. Populations of peripheral tissue dendritic cells, upon pathogen detection, become activated and migrate to T cell areas of draining lymph nodes (LNs) where they stimulate antigen-specific T cell responses to initiate immunity or tolerance. These are termed ‘migratory dendritic cells’ [2]. In addition to their ‘educator’ role, dendritic cells can perform as effector cells, with the ability to secrete cytokines and growth factors to manipulate the tissue environment [3]. Although a prominent role of dendritic cells is the polarization of naïve T cells, they are also able to interact with other immune cells including B and natural killer (NK) lymphocytes and innate lymphoid cells (ILCs) [4].

## DENDRITIC CELLS AS A DISTINCT HEMATOPOIETIC LINEAGE

**FIGURE 1:**
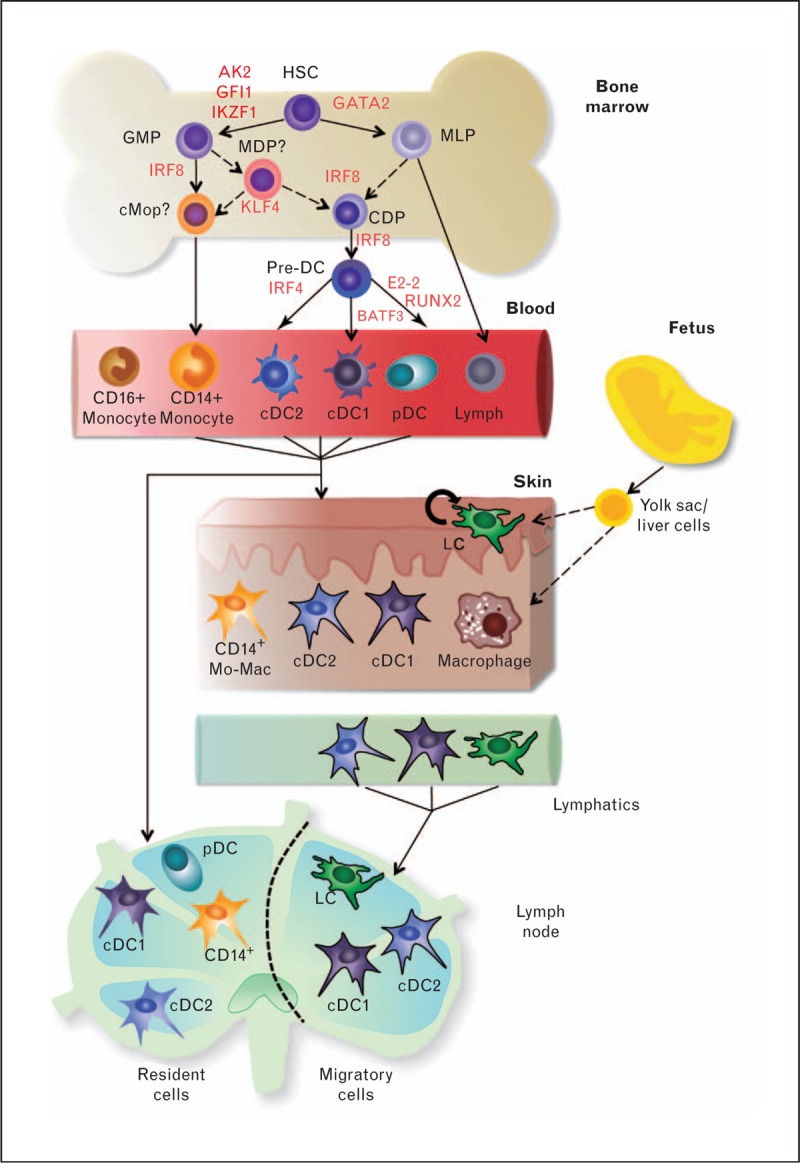
Human dendritic cell subsets and their ontogeny. Human dendritic cells are derived from hematopoietic stem cells in the bone marrow through a series of as yet undefined progenitors and precursors under the control of specific transcription factors. In the mouse model, dendritic cell differentiation occurs through the macrophage and dendritic cell precursor (MDP) and subsequently common dendritic cell precursor (CDP) and predendritic cell, but this path is as yet undefined in humans. Differentiated monocyte and dendritic cell subsets circulate in peripheral blood and can be found as resident cells in lymphoid tissue. In skin, monocyte-derived cells, cDC1, cDC2, tissue macrophages, and lymphoid cells can be found. On activation, cDC1, cDC2, and lymphoid cells can migrate via lymphatics to draining lymph nodes where they are distinguishable from resident cells. AK2, adenylate kinase 2; BATF3, basic leucine zipper transcriptional factor ATF-like 3; cMoP, common monocyte progenitor; E2-2/TCF4, transcription factor 4; GFI1, growth factor independent 1; GMP, granulocyte macrophage progenitor; HSC, hematopoietic stem cell; IKZF1, Ikaros family zinc finger 1; KLF4, Kruppel-like factor 4; MLP, multipotent lymphoid progenitor.

Based on in-vitro observations [5], it was previously thought that dendritic cells were derived from monocytes. However multiple lines of evidence indicate that, at least in the steady state, dendritic cells arise through a dedicated pathway of differentiation [6] (Fig. 1). In human gene expression studies, dendritic cells form a separate cluster from monocytes and macrophages [7,8] and lineage tracing in mice confirms their distinct identities [9]. This distinction breaks down in inflammation when inflammatory dendritic cells arise from monocytes [10]. It is likely that in-vitro monocyte-derived dendritic cells (mo-DCs) are surrogates for these inflammatory dendritic cells.

## CLASSIFICATION OF DENDRITIC CELLS, MONOCYTES, AND MACROPHAGES

A recent consensus study [11] has defined the nomenclature of dendritic cells, monocytes, and macrophages, based on ontogeny (monocyte or dendritic cell lineage), anatomical location, and surface antigen expression: two myeloid (or conventional/classical) dendritic cell subsets are defined as: cDC1, expressing CD141/BDCA3, Clec9A, XCR1; and cDC2, expressing CD11c, CD1c/BDCA1, in humans. The third dendritic cell subset comprises plasmacytoid dendritic cells (pDCs) expressing CD123, CD303/BDCA2, and CD304/BDCA4. Human monocytes, representing 10–20% of PBMC, are defined by their relative expression of CD14 and CD16 as classical monocytes (CD14+CD16−), intermediate monocytes (CD14+CD16+), and nonclassical monocytes (CD14lowCD16+) (Fig. 1). All dendritic cells and monocytes are found in the peripheral blood. In non-lymphoid tissues (e.g. epithelia), there are usually cDC1 and cDC2, but no pDCs in the steady state [12]. In lymph nodes, both migratory and resident dendritic cell subsets are found, including pDCs [2]. Most tissues contain macrophage populations composed of both monocyte-derived cells, with a rapid turnover, and fixed macrophages that persist for many years, independently of the bone marrow. RUNX3+ cells of the epidermis are also self-renewing and capable of surviving independently of the bone marrow. However, unlike fixed macrophages, lymphoid cells are able to leave the epidermis and mature into dendritic cells that enter the T cell zones of LNs (Fig. 1). Macrophages are CD11c negative, gain expression of CD163, LYVE-1, and FXIIIa, and are autofluorescent because of accumulation of pigment such as lipofuscin and melanin granules.

## GENETIC CONTROL OF DENDRITIC CELL DEVELOPMENT AND FUNCTION

**FIGURE 2:**
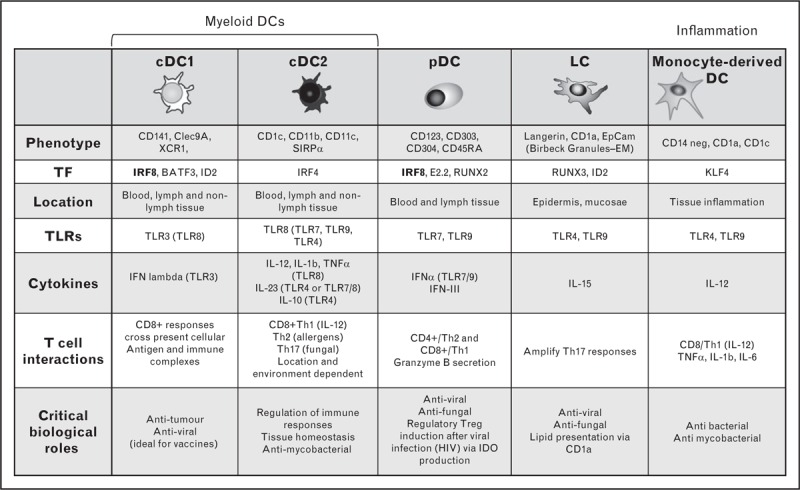
Phenotype and function of human dendritic cell subsets. Characteristic phenotype, transcription factor requirements, Toll-like receptor (TLR) expression, cytokine production, T cell interactions, and biological roles of human dendritic cell subsets. IDO, indolamine 2,3-dioxygenase; RUNX2/3, runt-related transcription factor 2/3; SIRPα, signal regulatory protein alpha.

Dendritic cells and monocytes develop in the bone marrow under the control of distinct transcription factors. They develop discrete functions owing to their expression of particular pattern recognition receptors (PRRs) and elaboration of specialized secretory products (Fig. 2).

cDC1 are dependent on BATF3 and IRF8 for development [13], have superior cross-presenting ability via Clec9a and secrete TNFα, IFNγ and IFNλ more abundantly than IL-12p40 on TLR3 ligation [14–16].

cDC2 require IRF4 and KLF4 expression for differentiation [17,18]. They express TLR1, 2, 4, 5, and 8, produce IL-12 [19] to prime CD4+ Th1 cells, proinflammatory cytokines IL-1β, TNFα, IL-23 and anti-inflammatory IL-10, depending on their location and environment. They variably possess many other functional receptors (e.g. Clec7A, Clec6a, CD205) and can generate Th17 or Th2 responses to fungal antigens or allergens, respectively [20]. Tissue cDC2 can detect glycolipid antigens of mycobacteria through CD1a expression [21].

pDCs are dependent on transcription factors RUNX2, E2-2, BCL11A, IRF7, and IRF8 [22–24]. Through TLR7 and TLR9 ligation, they are the major IFNα producing cells in response to viral infection. pDC can activate both Th1 and Th2 CD4^+^ responses and may induce Treg differentiation after viral infection and in the thymus [25].

Langerhans cells are dependent on RUNX3 and ID2 and require IL-34 and TGFβ for development [26,27]. In inflammation, lymphoid cells can respond to selected intracellular pathogens and viruses and migrate to T cell areas of draining LNs under the influence of TNF and IL-1β [28] and secrete IL-15. They appear to maintain tolerance to commensals through presentation of antigen in the context of CD1a and Treg induction [29].

mo-DCs are found in inflammatory tissues. In response to microbial challenge, monocytes downregulate CD14, upregulate dendritic cell markers including CD1a and CD1c, and are able to activate naïve T cells. In tissues, they synthesize inflammatory cytokines including IL-12, TNFα, IL-1β, IL-6, IL-23, inducible nitric oxide synthase (iNOS) [30] and stimulate Th1, Th2, or Th17 responses [31].

## DENDRITIC CELLS IN PRIMARY IMMUNODEFICIENCY DISEASE

Dendritic cells may be reduced in number, defective in function, or both (Table 1). Three syndromes associated with dendritic cell and monocyte deficiency have been identified by profiling patients with unexplained immunodeficiency. Heterozygous mutations in GATA2 constitute the largest group of cases and patients often survive undetected into adulthood [32–34]. Biallelic IRF8 deficiencies present at a young age with susceptibility to infection and myeloproliferation, similar to the IRF8-knock-out mouse, whereas mono-allelic IRF8 mutation was identified in an adult cohort with mycobacterial disease and represents a more subtle hypomorphic phenotype [35].

**Table 1:** Dendritic cell (DC) deficiency and dysfunction in primary immunodeficiency disease (PID)

	Gene	Clinical phenotype	Cell phenotype	DC
Deficiencies	Bi-allelic IRF8	Mycobacterial and viral infection; intracerebral calcification and developmental delay	Loss of all monocyte and DC subsets. Myeloproliferation	Complete absence of DC/monocyte but preservation of tissue macrophages and lymphoid cells
	GATA2	Mycobacterial, viral (HPV) infection. Lymphedema, deafness, autoimmunity, malignancy, MDS/AML	Dendritic cell, monocyte, B and NK lymphoid (DCML) deficiency	Complete absence of DC/monocyte but preservation of tissue macrophages and lymphoid cells
Pancytopenias	AK2 (reticular dysgenesis)	Neonatal fatal septicemia; hypoplasia of lymphoid organs	Global leukocytopenia	Global loss of monocytes, DCs and lymphoid cells
	CXCR4 (WHIM)	Warts (HPV), recurrent bacterial infections, carcinomas	B cell lymphopenia (hypogammaglobulinemia), myelokathexis with neutropenia	Reduced numbers of monocytes and DCs
Dysfunction	MHC class II CIITA, RFXANK, RFX5, RFXAP	Failure to thrive, diarrhea, respiratory tract infections, liver/biliary tract disease	Loss of MHC class II expression on leukocytes	Deficient antigen presentation and failure to mount effective CD4+ T cell responses
	WASp (Wiskott-Aldrich Syndrome)	Thrombocytopenia, bacterial and viral infections, atopia, autoimmunity, IgA nephropathy, lymphoma	Progressive reduction in T cells	Cytoskeletal protein; affects DC migration and immune synapse with T cells. Impaired T cell and antibody responses
	CD40/CD40L	Opportunistic infections; gastrointestinal and liver/biliary tract disease	Defective class switching: IgM+ and IgD+ B cells only; neutropenia	Impaired DC signaling cytokine production and cross-presentation
	TCF4/E2-2 (Pitt-Hopkins Syndrome)	Recurrent infections in 35% of patients. Distinct facial features, epilepsy, intellectual disability	Low IgM	Impaired pDC IFNα responses *in vitro*
	STAT3 (Hyper IgE)	Bacterial (*S. aureus*), *Aspergillus*, *Pneumocystis jirovecii*, mucocutaneous candidiasis, distinctive facial features	Reduced B cells, elevated IgE with decreased specific antibodies	Impaired IL-10 responses in DCs
	IRF7	Severe influenza infection in childhood	No defects reported	Impaired IFN types I and III production from pDC

Table detailing PID syndromes for which there is evidence for DC deficiency or dysfunction, together with the causative gene, predominant clinical phenotypes, cell phenotype, and DC abnormality described. AK2, adenylate kinase 2; CXCR4, CSC-chemokine receptor 4; GATA2, GATA binding protein 2; IRF, interferon regulatory factor; NK, natural killer; pDC, plasmacytoid dendritic cell; STAT3, signal transducer and activator of transcription 3; TCF4/E2-2, transcription factor 4; WHIM, warts, hypogammaglobulinemia, immunodeficiency, and myelokathexis.

### GATA2 mutation

Constitutive heterozygous GATA2 mutation causes a complex disorder with a wide spectrum of age and phenotype at presentation and is associated with several clinically described syndromes including Emberger syndrome (primary lymphedema and MDS/AML), familial MDS/AML, and MonoMac (monocytopenia with mycobacterium avium complex). Extra-hematopoietic effects include congenital deafness, primary lymphedema, autoimmunity, and HPV-driven malignancies. In the hematopoietic compartment, loss of peripheral blood and tissue mononuclear cells (dendritic cell, monocyte, B and NK cells deficiency; DCML deficiency), associated with a high serum Flt3 ligand, is an almost universal finding at presentation [36] although de-novo acute myeloid leukaemia (AML) can develop without prior clinical immunodeficiency [34]. Interestingly, tissue macrophages and epidermal lymphoid cells are relatively preserved, in keeping with their independence from blood borne precursors [37]. Presentation of GATA2 mutation in childhood is often related to myelodysplasia (MDS)/chronic myelomonocytic leukaemia (CMML), (particularly associated with monosomy 7), although prior or concurrent immunodeficiency features may be elicited [38^▪▪^]. In adults, primary immunodeficiency may be a more prominent feature with susceptibility to mycobacterial, viral (human papillomavirus) and fungal infection, autoimmunity, and respiratory compromise due to recurrent bacterial infections and/or pulmonary alveolar proteinosis. Development of MDS/AML (in approximately 50% of patients) is associated with the acquisition of additional cytogenetic (monosomy 7/8) or molecular genetic abnormalities [39] and development of additional cytopenias or CMML-like features. In patients who do not develop early MDS/AML, survival into adulthood is likely to be facilitated by immune memory developed by a relatively intact immune system in childhood with normal class-switched immunoglobulin, grossly intact T cell compartment, and normal responses to childhood vaccines. Although many GATA2 mutations have been described, there is little correlation between genotype and phenotype as members of the same family can be present with different clinical pictures. The influence of genetic background, effect of additional constitutive or acquired mutations, and environmental exposure on the pathogenesis and clinical phenotype are yet to be fully elucidated.

### Interferon regulatory factor 8 mutations

The IRFs are classic examples of single genes expressed in multiple functional gene sets as they are involved not only in interferon signaling, inflammation, and regulation of cytokine production but also have critical roles in hematopoietic cell differentiation [40,41]. IRF8 (interferon consensus sequence-binding protein; ICSBP) is necessary for the differentiation of all monocyte and dendritic cell subsets, with depletion of these subset equivalents in a mouse model bearing a targeted null allele [42–44]. The BXH2 mouse model, carrying a hypomorphic IRF8^R294C^ mutation [45] shows depletion of predominantly cDC1s and to a lesser extent pDCs [46]. Both models show impaired IL-12 production in the context of myeloproliferation.

IRF8 is a key player in the IFNγ/IL-12 axis [47], particularly important for resistance to mycobacterial infection [48]. It regulates many critical elements, including the differentiation of monocytes/dendritic cells, the production of IFNγ from T cells and IL-12 from myeloid cells, the transcriptional response to these cytokines, and the relevant polarization of T cells [49–51]. The central role of IRF8 illustrates the concept that single immune genes determine the response to specific classes of pathogen by coordinating multiple pathways in many different cell types.

Myeloproliferation observed in IRF8 deficiency likely results from a number of mechanisms including the failure to block the pro-granulopoietic effects of C/EBPα [52], and failure to terminate emergency hematopoiesis following an infectious stimulus [53].

In humans, biallelic IRF8 deficiency has been described in one individual with autosomal recessive K108E mutation, presenting at 10 weeks of age with disseminated BCG (Bacillus Calmette-Guérin) infection following vaccination, oral candidiasis, cachexia, and intracerebral calcification [35], subsequently associated with developmental delay. In keeping with IRF8 deficient mice, all monocyte and dendritic cell subsets were absent in the context of marked myeloproliferation. The severe immune defect necessitated stem cell transplantation, which resulted in complete donor cell repopulation of the APC compartment, in blood and tissues, and resolution of BCG infection and myeloproliferation.

Autosomal dominant T80A mutation was identified from a cohort of adult patients with mycobacterial infection. Analysis of the monocyte/dendritic cell compartment revealed a subtle defect in CD1c expression on cDC2s, which is at odds with the relative dependence of cDC1 and pDC on IRF8 in mouse. This contradiction may be related to differences in the specific mutation in IRF8, or divergence in function of apparently homologous subsets between the two species. In both clinical cases, mycobacterial infection responded to chemotherapy, life expectancy was not affected, and stem cell transplantation was not required.

### Quantitative dendritic cell deficiency in other known disorders

Reticular dysgenesis, due to mutations in adenylate kinase 2 (AK2), results in severe pancytopenia which includes monocytes, dendritic cells, and lymphoid cells [54–56].

WHIM (warts, hypogammaglobulinemia, immunodeficiency, and myelokathexis) presents as a progressive immunodeficiency. CXCR4 mutation (CSC-chemokine receptor 4) [57] prevents leukocytes leaving the bone marrow resulting in neutropenia and deficiency, but not absence, of monocytes and dendritic cells [58].

Ikaros/IKZF1 mutation in a single reported case describes a severe pancytopenia affecting monocytes and probably dendritic cells [59]. However, a recent paper [60] describes 29 individuals from six families with heterozygous IKZF1 mutations and a predominant phenotype of low serum immunoglobulin and progressive B cell loss without overt pancytopenia. As IKZF1 is required for pDC differentiation in mouse [61], these cases may reveal more subtle defects in dendritic cells.

Neutropenic states involve genes implicated in dendritic cell development in mice, including *GFI1*[62] and *ELANE* (congenital and cyclical neutropenia) [63] but the effect of these mutations on dendritic cells has not yet been assessed.

### Defects of dendritic cell function

Bare lymphocyte syndrome, caused by mutations in CIITA, RFXANK, RFX5, or RFXAP [64], is a severe immunodeficiency due to lack of MHC class II expression characterized by recurrent bacterial infection, chronic viral infections, failure to thrive, and death before the age of 10 years. Deficient Ag presentation leads to reduced numbers of CD4+ T cells and failure to mount effective CD4+ T cell responses or make immunoglobulin in response to vaccines [65].

Wiskott-Aldrich syndrome (WAS), caused by mutations in a cytoskeletal protein, WAS protein (WASp), affects dendritic cell migration and immune synapse formation with T cells [66] but may enhance dendritic cell cross-presentation [67]. Impaired T cell and antibody responses result in the risk of recurrent severe infection. Additional features include atopia, autoimmune hemolysis, vasculitis, and thrombocytopenia [68].

CD40/CD40L deficiency results in defective isotype switching and impaired dendritic cell signaling, cytokine production, and cross-presentation [69,70].

Pitt-Hopkins Syndrome, associated with mutations in the transcription factor E2-2 (TCF4), although not classified as PID, impairs in-vitro responses of pDCs [23]. A recent study [71] revealed recurrent infection in 35% of patients. Additional features include typical facies, intellectual disability, and epilepsy.

STAT3 mutation (signal transducer and activator of transcription 3) in humans is not associated with numerical loss of dendritic cells but responses to IL-10 are impaired [72].

IRF7 mutation, recently described in one compound heterozygous patient, results in severe influenza infection, attributed to impaired IFN type I and III production by pDCs [73^▪▪^].

## ANALYSIS OF DENDRITIC CELLS IN PRIMARY IMMUNODEFICIENCY DISEASE

The potential of dendritic cell and monocyte analysis to assist in the clinical diagnosis and understanding of PID is only just being realized [74]. Simplistically speaking, the hematopoietic origin of dendritic cells lies between lymphoid and myeloid cells and dendritic cell number and function may be perturbed in association with defects of either lineage. Identifying perturbations in the dendritic cell/monocyte compartment may help to confirm the existence of an immune dysfunction, understand a phenotype, predict response to vaccination, or guide the analysis of next generation sequencing investigations.

Diagnosis of known dendritic cell deficiency syndromes: Myeloproliferation of biallelic IRF8 deficiency may mask the absolute monocytopenia on automated blood counters but absence of dendritic cells and monocytes in this context is, to date, pathognomonic. Depth of mononuclear cell loss, together with increase in serum Flt3L, in GATA2 mutation may inform ‘stage’ of disease and associate with development of symptoms or progression to bone marrow failure.

Discovering novel dendritic cell deficiency syndromes: Analysis of the recently described ‘human gene connectome’ (HCG) [75] has identified novel candidate PID-causing genes based on their biological proximity to known causative genes [76^▪^]. This work predicts that PID may be caused by mutations in many genes related to dendritic cell differentiation or function including KLF4, ID2, IRF4, RUNX1, RUNX2, and TLRs.

Understanding dendritic cells in known primary immunodeficiency diseases: Dendritic cell deficiency or dysfunction may contribute to the diagnosis and phenotype of many known PIDs, in which functions of the mutated gene have not yet been explored beyond lymphocytes, for example TLR3 [16], TRAF3 [77], and ADA [78].

### A practical approach to analysis

**FIGURE 3:**
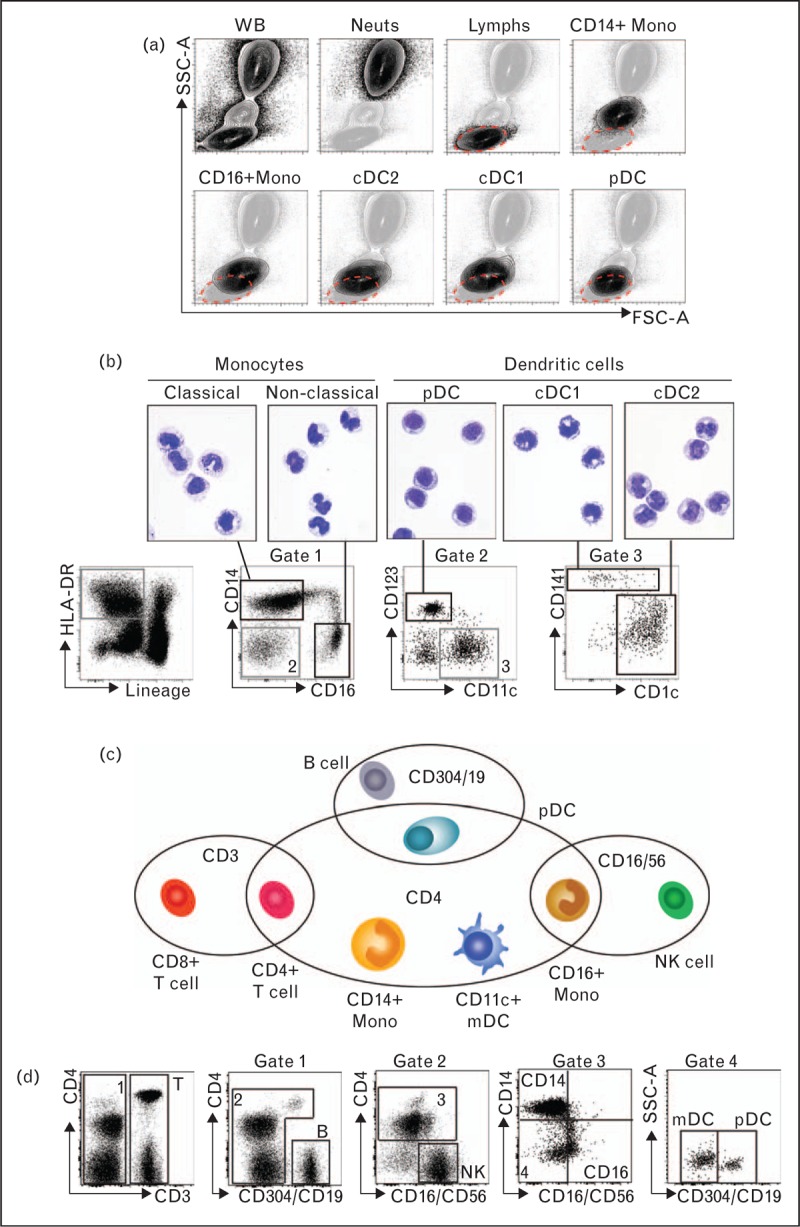
Human blood monocyte and dendritic cell analysis. (a) Flow cytometric analysis of whole blood (WB) showing forward and side scatter properties of leukocytes (black contours) superimposed against whole blood cells (underlying gray contours). The dashed gate indicates where lymphocytes fall on the 2D plot, illustrating that some nonclassical monocytes and dendritic cells fall predominantly within this gate. Lymphs, CD3+ lymphocytes; Mono, monocytes; Neuts, CD16+ neutrophils. (b) 8-color (10-channel) flow cytometry to identify monocyte and dendritic cell populations in blood, together with their morphological appearance after fluorescence activated cell sorting (FACS), cytospin, and Giemsa staining. (c) Diagrammatic representation of adapted lymphocyte subset panel utilizing CD4 expression on APCs to distinguish them from lymphocytes when APC/lymphocyte antigens are combined in one fluorescent channel. (d) Flow cytometric gating strategy to identify T, B, and NK lymphocytes alongside classical and nonclassical monocytes.

Dendritic cells are too rare to enumerate morphologically by cytological examination of whole blood and automated blood counters are unable to distinguish dendritic cells from lymphocytes by light scatter properties (Fig. 3a). Classical, but not nonclassical, monocytes can be identified morphologically and on automated counters but monocytopenia can be masked by the appearance of immature myeloid cells with similar light scatter properties. As such, ‘first line’ hematological investigations are unable to identify dendritic cell deficiency and may underestimate monocytopenia.

Flow cytometry lends itself well to clinical dendritic cell/monocyte analysis as it allows accurate identification and enumeration of rare populations of cells from clinically appropriate volumes of blood. A minimum of six channels is required to identify all monocyte and dendritic cell subsets, including human leukocyte antigen-DR (HLA-DR) expressed on all APCs, lymphocyte lineage markers to exclude HLA-DR+ T-, B-, NK-cells, and CD34+ progenitor cells, CD14 and CD16 for monocyte analysis, CD123 or CD303/CD304 for pDC, CD141 or Clec9A for cDC1, and CD1c/BDCA1 for cDC2 (Fig. 3b). With a view to minimizing sample volumes and costs, a protocol has been devised to combine dendritic cell analysis with standard lymphocyte subset enumeration panels (Fig. 3c and d). This panel relies on the universal expression of CD4 by APCs, allowing segregation of dendritic cell/monocytes from lymphocytes using standard markers alone (CD3−, CD4+ cells). Further refinement is possible by combining one lymphocyte and one APC antibody in a single fluorescence detection channel to pull out monocyte subsets and to separate pDCs from cDCs [79].

Functional analysis poses more significant problems, given the broad array of pathogen receptors, costimulatory molecules, and potential cell outputs of different dendritic cell subsets, such that it is unlikely to be possible to interrogate all aspects of dendritic cell function in a single reaction. Whole blood cytokine analysis is well described, particularly for investigating the IL-12/IFNγ axis [80] but cell-specific dendritic cell/monocyte functional analysis is currently confined to the research laboratory.

### Whom to test

Ideally, patients with a high clinical index of suspicion for a dendritic cell deficiency would be selected for testing. However there are a number of obstacles to this approach.

First, given the complexities of the integrated immune system, it may not be possible to closely define this clinical category. Some (nonspecific) features of global monocyte and dendritic cell loss can be inferred from IRF8 and GATA2 mutations and these would include susceptibility to tuberculosis and atypical mycobacteria, viral infection (particularly HPV and herpes simplex virus), pulmonary pathology (recurrent viral and bacterial infection, and pulmonary alveolar proteinosis), and autoimmunity. Specific features of these mutations may also be sought, such as myeloproliferation in biallelic IRF8 mutation and MDS or extrahematopoietic features in GATA2 mutation.

Second, it is difficult, at present, to define the pretest risk without a better picture of the prevalence, which will only be achieved through much greater uptake of dendritic cell and monocyte enumeration by diagnostic laboratories.

We suggest, therefore, that a survey level analysis (six-color flow cytometry) should be performed in all new patients with suspected immunodeficiency alongside, for example, T and B cell subset analysis. An abnormal screen or a high index of suspicion should prompt full immunophenotyping of myeloid mononuclear cells.

This will facilitate the discovery of novel dendritic cell and monocyte disorders as well as build a picture of the dendritic cell/monocyte landscape in PID specifically and in immunity generally.

## CONCLUSION

Mouse models have suggested that dendritic cells are critical to initiate and maintain immunity and tolerance, yet little attention has been paid to their contribution in PID. Recent descriptions of dendritic cell deficiencies and dysfunctions in the context of immunodeficiency have highlighted their importance in this field. Clinical level screening for dendritic cell and monocyte defects will allow the identification of novel disorders and aid the diagnosis and pathophysiological understanding of immunodeficiency.

From a research perspective, through the coordinated investigation of dendritic cells in patients with immunodeficiency, the PID community is in a unique position to advance our understanding of the function of human dendritic cells and monocytes *in vivo*, looking to harness their immune potential for therapeutics across wider fields of medicine.

Aligning these efforts with those of next generation sequencing consortia such as the NIHR BRIDGE Project (PID) (https://bridgestudy.medschl.cam.ac.uk/pid.shtml) and 100,000 Genome Project (https://www.genomicsengland.co.uk), with the support of cohesive research networks such as the Genomics England Clinical Interpretation Partnership (GeCIP), will likely yield fruitful results.

## Acknowledgements

The authors would like to thank Newcastle upon Tyne Hospitals NHS Foundation Trust Paediatric Immunologists and Blood Sciences laboratory (flow cytometry) for their vital contributions to this work.

### Financial support and sponsorship

This work was supported by Wellcome Trust (V.B.), BrightRed (V.B., M.C.), and BloodWise (M.C.).

### Conflicts of interest

There are no conflicts of interest.

## REFERENCES AND RECOMMENDED READING

Papers of particular interest, published within the annual period of review, have been highlighted as:

▪ of special interest
▪▪ of outstanding interest
